# Responses of Tectal Neurons to Contrasting Stimuli: An Electrophysiological Study in the Barn Owl

**DOI:** 10.1371/journal.pone.0039559

**Published:** 2012-06-20

**Authors:** Yael Zahar, Hermann Wagner, Yoram Gutfreund

**Affiliations:** 1 The Department of Physiology and Biophysics, The Ruth and Bruce Rappaport Faculty of Medicine, Technion, Haifa, Israel; 2 Institute of Biology II, RWTH Aachen, Aachen, Germany; Imperial College London, United Kingdom

## Abstract

The saliency of visual objects is based on the center to background contrast. Particularly objects differing in one feature from the background may be perceived as more salient. It is not clear to what extent this so called “pop-out” effect observed in humans and primates governs saliency perception in non-primates as well. In this study we searched for neural-correlates of pop-out perception in neurons located in the optic tectum of the barn owl. We measured the responses of tectal neurons to stimuli appearing within the visual receptive field, embedded in a large array of additional stimuli (the background). Responses were compared between contrasting and uniform conditions. In a contrasting condition the center was different from the background while in the uniform condition it was identical to the background. Most tectal neurons responded better to stimuli in the contrsating condition compared to the uniform condition when the contrast between center and background was the direction of motion but not when it was the orientation of a bar. Tectal neurons also preferred contrasting over uniform stimuli when the center was looming and the background receding but not when the center was receding and the background looming. Therefore, our results do not support the hypothesis that tectal neurons are sensitive to pop-out per-se. The specific sensitivity to the motion contrasting stimulus is consistent with the idea that object motion and not large field motion (e.g., self-induced motion) is coded in the neural responses of tectal neurons.

## Introduction

In pop-out stimuli multiple objects are scattered in a visual scene in which one, the target, is different from the remaining objects, the distracters, by one sensory feature only. In such conditions the target tends to “pop-out” when human subjects are tested; i.e., it is easily identified and the time for identification is largely independent of the number of distracters [1], [2]. The psychological phenomenon of pop-out is directly related to the more general behavior of visual search. In visual search animals overtly or covertly shift attention to salient parts of the visual scene [3]. Thus, the visual system has the property to rapidly identify and select salient features or objects [4].

In a recent study of visual search in free-viewing barn owls it was demonstrated that barn owls confronted with contrasting scenes in which one object was differently oriented, looked earlier, longer and more often at the odd object compared to the distracters [5]. This result demonstrates that stimuli that are oriented differently from the background are perceived as salient in barn owls as in humans and suggest similarities of visual search strategies between these distinct species. The neural mechanisms that allow the rapid selection of odd targets in orientation or in any other sensory feature remain elusive.

The superior colliculus (SC) plays an important role in visual search [6]–[8]. The response of many SC neurons to stimuli within their classical receptive field (RF) is attenuated by the existence of competing distant stimuli [9]. This competitive interaction has been studied, among others, in the optic tectum of barn owls (OT, the avian homologue of the superior colliculus) [10], [11]. It has been suggested that competitive interactions are mediated by a GABAergic midbrain nucleus (Imc, nucleus istmi pars magnocellularis) that mediates global inhibition across the map of space in the OT giving rise to competitive interactions between signals from simultaneously presented stimuli [12]–[14]. While the model proposed by these authors explains global spatial interactions, it cannot explain, by its own, competitive interactions that are specific to a stimulus feature (such as orientation, velocity, etc.). Such feature specific interactions are required for the preferred response to a “pop-out” stimulus.

In this paper we use the term contrasting stimulus to refer to a stimulus that differs in one feature from a uniform background (as in classical pop-out scenes). We studied three different features (motion, orientation, looming). We asked whether tectal neurons are selectively responsive to contrasting stimuli compared to uniform stimuli. The motivation to search for such selectivity in the OT and not elsewhere in the barn owl's brain lies in the emerging notion that the OT is directly involved in the selection of the most salient target for the focus of attention [15]–[17]. Our results demonstrate sensitivity to contrasting stimuli in horizontal motion but not in orientation or looming.

## Materials and Methods

### Ethics Statement

Five barn owls (*Tyto alba*) were used in this research. All owls hatched in captivity and were raised and kept in a large flight cage equipped with perching spots and nesting boxes. The owls were provided for in accordance with the guidelines of the Technion Institutional Animal Care and Use Committee. The protocol was approved by the Committee on the Ethics of Animal Experiments of the University of the Technion (Permit Number: IL-087-07-10). All surgery was performed under isoflurane anesthesia, and all efforts were made to minimize suffering. In all recording sessions the animals were sedated with nitrous oxide. During recording sessions no painful procedures were carried out.

### Surgical Procedures

Owls were prepared for repeated electrophysiological experiments in a single surgical procedure: the owl was anesthetized with isoflurane (2%) and Nitrous Oxide in Oxygen (4∶5). The scalp was prepared with Betadine surgical scrub. An incision was made in the scalp and the skull was scrapped clean in two places. At one place, just anterior to the neck muscle, a small stainless steel plate with a protruding bolt was cemented to the skull using dental cement. At a second place, determined by stereotaxic coordinates, a craniotomy was performed and a threaded recording chamber was cemented to the skull. The wound was sutured, and incisions were infused with Lidocaine. After surgery the animal was left to recover over-night in an individual cage and then released back to its home cage.

### Electrophysiological recordings

Before each electrophysiological session the owl was moved to an individual cage and deprived of food over-night. At the beginning of the recording session the owl was anesthetized briefly with isoflurane (1.5%) and Nitrous Oxide in Oxygen (4∶5), wrapped in a soft leather jacket and placed in a stereotaxic apparatus at the center of a double wall sound-attenuating chamber (internal size – 2.05×1.7×1.95m) lined with echo suppressing foam. The head was bolted to the stereotaxic apparatus and aligned using retinal landmarks (as described in [17]). Once the bird was secured in place, isoflurane was removed from the gas mixture, and the bird was maintained on a fixed mixture of Nitrous Oxide and Oxygen (4∶5). The head chamber was opened, and a glass-coated tungsten micro-electrode was driven into the recording chamber using a motorized manipulator. Since eye movements in barn owls are limited to a range that is smaller than ±3° [18], we did not immobilize or control for eye movements. Electrophysiological recordings began not earlier then 1 hour after removal of isoflurane, allowing enough time to recover from the anesthetic agent. A Tucker-Davis Technologies System3 and an on-line spike sorter (MSD, Alpha-Omega Inc, Nazareth, Israel) were used to record and isolate action potentials from single neurons or a small cluster of neurons (multiunit recording). Multiunit recordings were obtained by manually setting a threshold consistently selecting the largest unit waveforms in the recorded site. Single units were isolated using a template-based sorting. Because of the characteristic bursty firing of many tectal sites, adequate template matching was difficult to obtain. Therefore most of the recordings in our data set were considered multiunit recordings. At the end of each recording session the chamber was treated with chloramphenicol ointment (5%) and closed. The owl was then returned to its home flying cage.

### Targeting of the OT

The identification of the OT was based on stereotaxic coordinates and expected physiological properties. The OT was recognized by characteristic bursting activity and spatially restricted visual receptive fields [19]. After reaching the most superficial bursty layer the electrode was advanced 300 µ deeper before recording began. Recording sites, spanned both bursty, and non-bursty layers [19], [20], were separated by at least 300 µ along the penetration tracks. In an initial analysis, PSTHs were drawn separately for bursty layers population and non-bursty layers population. No qualitative differences were observed between the two populations. Therefore, all recording sites were combined to obtain the population responses

### Visual stimuli

**Figure 1:**
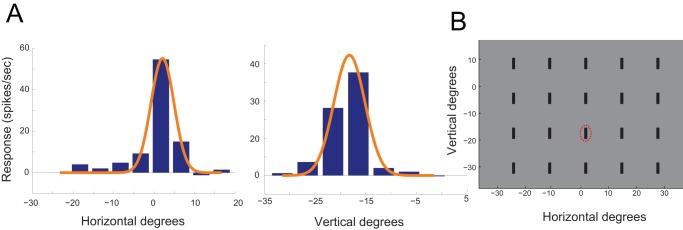
The process of positioning the center stimulus and background elements in the visual display. A) The neural response of a single recording site in the OT to a vertical bar as a function of the horizontal position of the bar. The red curve is the Gaussian fit to the data points. B) The neural response of the same recording site to the vertical position of the bar. Format as in A. C) Graph showing a typical display on the screen. The x-axis is the horizontal position (in degrees) and the y-axis is the vertical position (in degrees) relative to the visual axis. The red ellipse designates the visual receptive field of the site shown in A and B. The center bar (inside the RF) is shown together with the background bars.

The visual stimuli were computed with Matlab, using the Psychophysics Toolbox extensions [21] and projected (refresh rate 72 Hz, XD400U; Mitsubishi) on a calibrated screen inside the sound attenuating chamber (screen size 150 cm ×115 cm, 1.5 meter away from the owl). The projector was positioned outside the chamber, projecting the image through a double glass window. All locations are given in azimuth relative to the owl's midsagital plane and elevation relative to its horizontal visual plane. Visual stimuli where dark bars or dots presented on a gray background (luminance of background screen was 17 cd/m^2^ and luminance of bars and dots was 8–12 cd/m^2^). In each recording site we first estimated the visual RF by manually projecting a visual stimulus on the screen (a dark dot about 1^o^ in diameter). Then, the horizontal and vertical tuning curves were recorded by varying the azimuth and elevation of a vertical bar (length 4^o,^ width 1°) on the screen at the fixed elevation or azimuth of the estimated RF, respectively (Fig. 1A). At each location the bar was flashed on the screen for 200 msec. The locations were randomly interleaved in stimulus sets that were repeated 10–20 times.

After mapping the RF a test paradigm was applied, corresponding to one of the following pairs of stimuli: 1) Motion stimuli, a vertical bar (length 4°^,^ width 1°) moving 1.9° to the left or 1.9° to the right. The duration of movement was 600 ms. In the motion stimulus, the bars were maintained displayed on the screen throughout the test. At time zero, the motion began for 600 ms then the display was frozen for another 500 ms after which the bars were shifted back to their initial locations and maintained static on the screen until the next stimulus. 2) Orientation stimuli, a tilted bar at either 45° or −45° from the vertical line (length of bar 4°). In the orientation stimuli, the bars appeared on the screen at time zero, were displayed for 600 ms and disappeared afterwards. 3) Looming stimuli, a dot with a diameter of 1° increasing its size to a diameter of 6° in 600 ms. The looming stimulus was paired with a receding stimulus of an initial dot of 6°diameter receding in 600 ms to a diameter of 1°. The looming/receding stimulus changed linearly in time. In the looming/receding paradigm the initial frame was displayed frozen for 2 s before the onset of the looming/receding motion. At the end of the motion the last frame was maintained for 500 ms after which all dots disappeared for 500 ms and reappeared with the initial conditions of the next stimulus. In the motion and looming paradigms the onset of the stimulus (time zero) was defined by the onset of movement and the stimulus was displayed on the screen before and after the movement. In the orientation paradigm no movement was involved and therefore the display was flashed on the screen at time zero and disappeared at the end of the stimulus.

In each test a center stimulus was positioned at the center of the RF surrounded by a rectangular array of background elements equally spaced in 10° intervals (Fig 1B). The center stimulus was displayed in three different conditions. In the singleton condition, only the center stimulus was presented, the background elements were maintained static in the motion and looming tests and not displayed in the orientation test. In the uniform condition, the center and background were moving or oriented identically. In the contrasting condition the center stimulus was different from the background elements, it was either oriented differently or moved in the opposite direction. In each test both types of stimuli served in interleaved trials as either a center stimulus or as background elements. All six conditions were randomly interleaved and repeated 15 times with an inter stimulus interval of 3 sec.

### Data Analysis

Unit responses to a visual stimulus were quantified as the number of spikes in a given time window after stimulus onset minus the number of spikes during the same amount of time immediately before stimulus onset (baseline activity). The duration of the time window for spike count was 800 ms starting from the onset of stimulation. The RF was defined using Gaussian fitting of the horizontal and vertical tuning curves (Fig 1A, red curves). The RF borders were defined as the values where the Gaussian curves reached 50% of their height. To observe the time course of the response, we generated PSTHs with 20 ms time bins. PSTHs were normalized to the maximum value achieved in each experiment and averaged across the population. The standard errors of the mean are depicted as the width of the PSTH curves. To quantify the contextual response we calculated the pop-out modulation index [40] as following: contrast index  =  (R_oddball_ – R_uniform_)/(R_oddball_ + R_uniform_), where R_oddball_ is the response to the center stimulus when it is different from the background elements, and R_uniform_ is the response to the same center stimulus when it is identical to the background elements. Positive values of this index indicate a preference for a center that is different from its background. For each recording site two indices were calculated; one when stimulus one was in the center and the other when its opposite stimulus was in the center. A Chi-square test was used to address the population distribution of the modulation indices in the following four categories: both indices are positive, both indices are negative, one is negative and two is positive; one is positive and two is negative. If the null hypothesis of equal distribution was rejected a further subdividing Chi-square analysis [22] was performed to address in which category the points tend to concentrate.

## Results

Responses of 109 recording sites in the OT were analyzed. Recording sites spanned both bursty layers (superficial and intermediate layers) (n = 75) and non-bursty layers (deep layers) [19] (n = 34). All recording sites were from the anterior part of the OT having visual receptive fields between left and right 20° and up and down 20° relative to the center of the visual field. The average RF size was 7.1±3° width by 6.8±2.7° elevation.

### Responses to orientation contrasting stimuli

**Figure 2:**
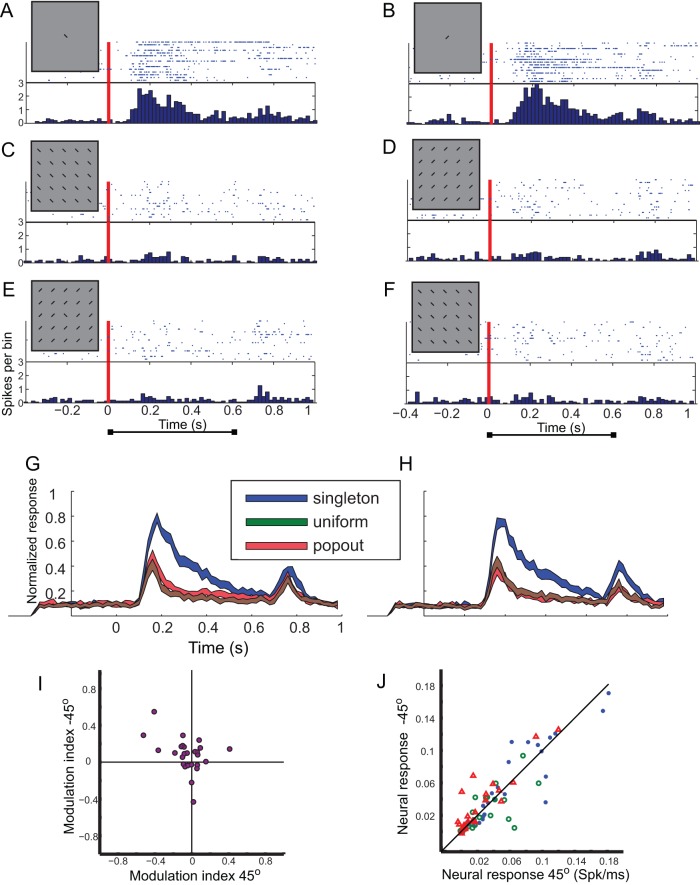
Summary of results from the orientation test. A–F) The responses of a single site to the orientation test. A) The raster plot shows responses to 15 repetitions of a single bar, oriented −45°, inside the RF (singleton condition) the inset demonstrates the visual stimulus. The corresponding PSTH is shown below the raster plot. The red vertical line designates the onset of stimulation, i.e., the appearance of the bar. B) Responses to 15 repetitions of a single bar inside the RF, oriented +45° (singleton condition). Format as in A. C) Responses to 15 repetitions of the center bar together with the background bars, all oriented −45°(uniform condition). Format as in A. D) Responses to 15 repetitions of the center bar together with the background bars, all oriented +45° (uniform condition). Format as in A. E) Responses to 15 repetitions of the center bar together with the background bars. The center is oriented −45° and the background bars +45° (contrasting condition). Format as in A. F) Responses to 15 repetitions of the center bar together with the background bars. The center is oriented +45° and the background bars −45° (contrasting condition). Format as in A. G) The PSTH curve of the population response to the singleton +45° (blue curve) is compared with the PSTH curve of the population response to the uniform +45° (green curve) and with the PSTH curve of the population response to the contrasting +45° (red curve). The width of the curves designate the SEM. H) The PSTH curve of the population response to the singleton −45° (blue curve) is compared with the PSTH curve of the population response to the uniform −45° (green curve) and with the PSTH curve of the population response to the contrasting −45° (red curve). The width of the curves designate the SEM. I) The scatterplot shows the modulation indices for the −45° center stimulus versus the +45° center stimulus. Each dot represents results from a single recording site. J) The neural responses to the −45° stimuli are shown versus the responses to the +45° stimuli. Each dot represents results from a single recording site. Blue symbols show results from the singleton conditions, red from the contrasting conditions and green from the uniform conditions. The diagonal line displays the locus of equal responses.

The orientation paradigm was tested in 26 recording sites. An example from a single recording site is shown in figure 2 A-F. In this site the neurons responded rigorously to the singleton stimuli of both orientations (Fig 2 A, B). The responses to the two orientations were not significantly different (*t*-test, p>0.05). The response of the same site to the oriented bars in the uniform condition was highly suppressed (t-test <0.01; Fig 2 C, D). The strong suppression of the responses was also observed in the contrasting condition where the bars in the RF were oriented differently than the bars outside the RF (t-test <0.01; Fig 2 E, F). Thus, this site demonstrated strong inhibition from the surround but no contrast sensitivity.

The result demonstrated in the above example was typical in the population of the recording sites. In all, the singleton conditions yielded the maximal responses and in 22 out of 26, the differences between the responses in the uniform and contrasting conditions were not significant (*t*-test; p>0.05). Figure 2G and H shows the population PSTHs obtained in the six conditions of the orientation paradigm. The strong contextual suppression of the responses can be clearly seen both in the uniform (red curves) and the contrasting (green curves) conditions. Figure 2I shows a scatterplot of the modulation indices obtained from the −45° orientation versus the modulation indices from the 45^o^ orientation. Dots appearing in the upper right quadrant indicate enhanced responses to contrasting stimuli compared to uniform. However, the dots were mostly close to zero and distributed in all four quadrates (Chi-square test, p>0.05) indicating similar responses to uniform and contrasting conditions. Figure 2H shows a scatterplot of the responses to the −45° orientation versus the responses to the 45° orientation, in the singleton (blue dots), uniform (green dots) and the contrasting (red dots) conditions. The dots are scattered evenly from both sides of the diagonal line (sign test, p>0.05) indicating that there is no general preference to one orientation versus the other. Only 5 sites out of 26 distinguished significantly between the two orientations in the singleton conditions and 3 sites in the non-singleton conditions (*t*-test, p<0.05). Thus, the tectal sites were mostly not tuned to the orientation of the bar.

### Responses to motion contrasting stimuli

**Figure 3:**
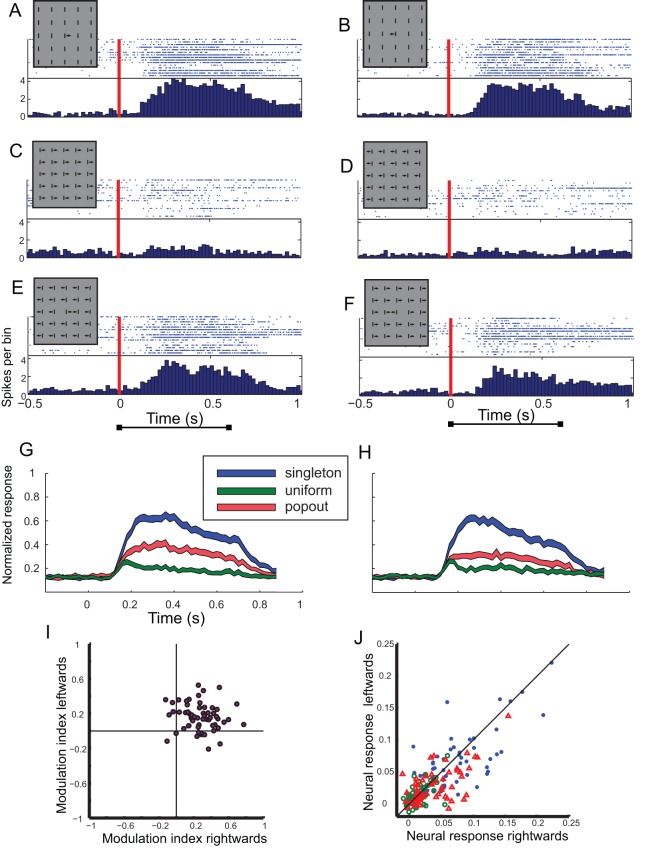
Summary of results from the motion test. A–F) The responses of a single site to the motion test. A) The raster plot shows responses to 15 repetitions of a bar inside the RF moving rightwards while the background bars are static (singleton condition). The inset demonstrates the visual stimulus. The arrow in the inset points to the direction of movement. The corresponding PSTH is shown below the raster plot. The red vertical line designates the onset of stimulation, i.e., the initiation of movement. B) Responses to 15 repetitions of a bar inside the RF moving leftwards while the background bars are static (singleton condition). Format as in A. C) Responses to 15 repetitions of the center bar moving rightwards together with all the background bars (uniform condition). Format as in A. D) Responses to 15 repetitions of the center bar moving leftwards together with all the background bars (uniform condition). Format as in A. E) Responses to 15 repetitions of the center bar moving rightwards together with background bars that are moving leftwards (contrasting condition). Format as in A. F) Responses to 15 repetitions of the center bar moving leftwards together with background bars that are moving rightwards (contrasting condition). Format as in A. G) The PSTH curve of the population response to the singleton rightwards movement (blue curve) is compared with the PSTH curve of the population response to the uniform rightwards movement (green curve) and with the PSTH curve of the population response to the contrasting rightwards movement (red curve). The width of the curves designate the SEM H) The PSTH curve of the population response to the singleton leftwards movement (blue curve) is compared with the PSTH curve of the population response to the uniform leftwards movement (green curve) and with the PSTH curve of the population response to the contrasting leftwards movement (red curve). The width of the curves designate the SEM. I) The scatterplot shows the modulation indices for the leftwards moving center stimulus versus the rightwards moving center stimulus. Each dot represents results from a single recording site. J) The neural responses to the leftwards moving center stimulus are shown versus the responses to the rightwards moving center stimulus. Each dot represents results from a single recording site. Blue symbols show results from the singleton conditions, red from the contrasting conditions and green from the uniform conditions. The diagonal line displays the locus of equal responses.

The motion paradigm was tested in 61 recording sites. An example from one site is shown in figure 3 A-F. In this example the moving bar in a singleton condition induced strong responses, independent of the direction of movement. The responses to the left direction were insignificantly different from the responses to the right (*t*-test, p>0.05, Fig 3A and B). The responses to the moving bars in the uniform condition were strongly suppressed compared to the singleton condition (Fig 3C and D), pointing again to strong inhibition from the surround. The responses to the moving bars in the contrasting condition (Fig 3E and F) were reduced from the singleton condition but were significantly stronger than in the uniform condition (t-test, p<0.01; Fig 3E and F compared to C and D). This tendency to respond preferentially to the contrasting stimuli compared to the uniform was apparent at the population level as well. In all, the singleton condition yielded the maximal average response and in 55 out of 61 sites the contrasting response was larger than the uniform response. Figures 3G and H compare the population responses to the moving bars in the singleton condition (blue), the uniform condition (green) and the contrasting condition (blue). It can be seen that the addition of background elements (uniform and contrasting conditions) reduced the population responses. However, this reduction was larger in the uniform condition compared to the contrasting condition. The scatter-plot of the modulation indices in the motion paradigm is shown in figure 3I. In this paradigm most of the dots were distributed significantly in the upper right quadrant (48 out of 61, Chi-square, p<0.01), indicating a selectivity for the motion contrasting stimulus over the uniform motion stimulus.

To address directional selectivity in the population of sites, we plotted for each recording site the responses to the left motion versus the responses to the right motion, in the singleton (blue dots), uniform (green dots) and the contrasting (red dots) conditions (Fig 3J). Out of 183 points 103 were below the diagonal line indicating a slight preference to rightward motion stimulus inside the RF. However this preference is not significant (sign test, p>0.05). Out of the 61 sites tested with the motion paradigm 27 sites distinguished significantly between the two directions of motion in the singleton condition (t-test, p<0.05) and 23 sites in the non-singleton conditions (*t*-test, p<0.05). However, it should be mentioned that the significant differences between responses to right versus left motions, observed in about half of the recording sites, maybe due to slight inaccuracies in the positioning of the bar at the center of the visual RF and not necessarily due to directional tuning of the neurons.

**Figure 4:**
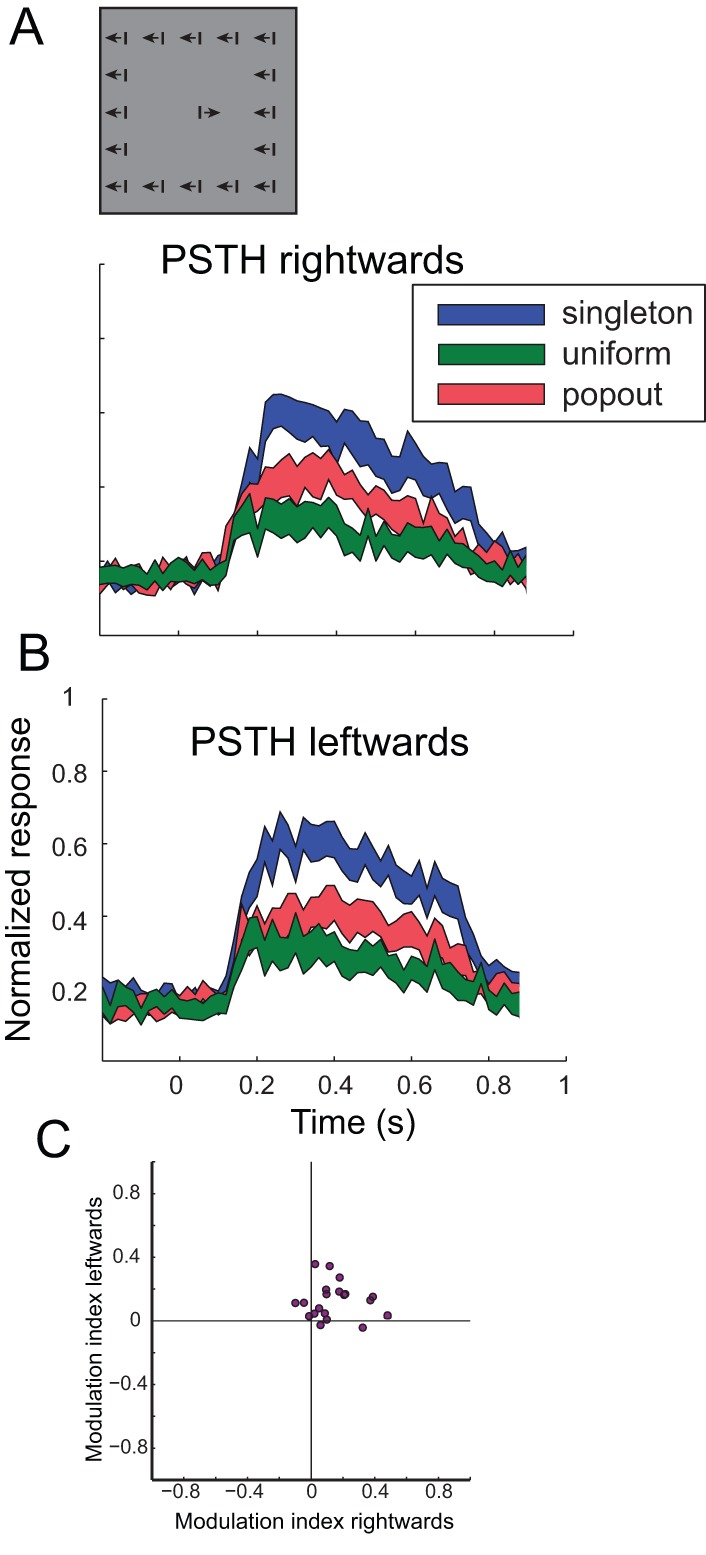
Responses to the motion test with large separation between center and background elements. A) The inset illustrates the test conditions where the stimulus at the center of the display is separated from the background elements by a gap of 20°. The PSTH curve of the population response to the singleton rightwards movement (blue curve) is compared with the PSTH curve of the population response to the uniform rightwards movement (green curve) and with the PSTH curve of the population response to the contrasting rightwards movement (red curve). The width of the curves designate the SEM. B) The PSTH curve of the population response to the singleton leftwards movement (blue curve) is compared with the PSTH curve of the population response to the uniform leftwards movement (green curve) and with the PSTH curve of the population response to the contrasting leftwards movement (red curve). The width of the curves designate the SEM. C) The scatterplot shows the modulation indices for the leftwards moving stimuli versus the rightwards moving stimuli. Each dot represents results from a single recording site.

One caveat in the motion paradigm is that in the pop-out condition the center bar is moving towards one of its neighbors and consequently the distance between the two bars is reduced from 10°to 6°. Therefore, the possibility that two bars appear simultaneously inside the RF is more likely in the contrasting condition compared to the uniform condition where the distance between bars is maintained at 10°. It is possible, therefore, that the observed stronger responses to contrasting stimuli are a result of this narrowing of the gap or some other sensitivity to local contrasts in or near the classical receptive field. To address this issue we performed a modified motion test in 20 tectal sites. In this test the close rectangle of background elements was omitted (see inset in Fig 4A). Thus, the background elements were positioned 20° from the center bar, making it unlikely that the response will be influenced by effects inside or near the RF. This modification of the paradigm did not change the basic result. The population response to the moving bars was larger in the contrasting condition compared to the uniform condition (Fig 4A and B, compare red curves with green curves). The scatter-plot of the modulation indices in the modified paradigm is shown in figure 4C. Most of the dots were, again, significantly distributed in the upper right quadrant (15 out of 20, Chi-square, p<0.01), indicating that the tendency for selectivity to motion contrast in tectal neurons is robust and does not depend on nearby elements.

### Responses to looming contrasting stimuli

**Figure 5:**
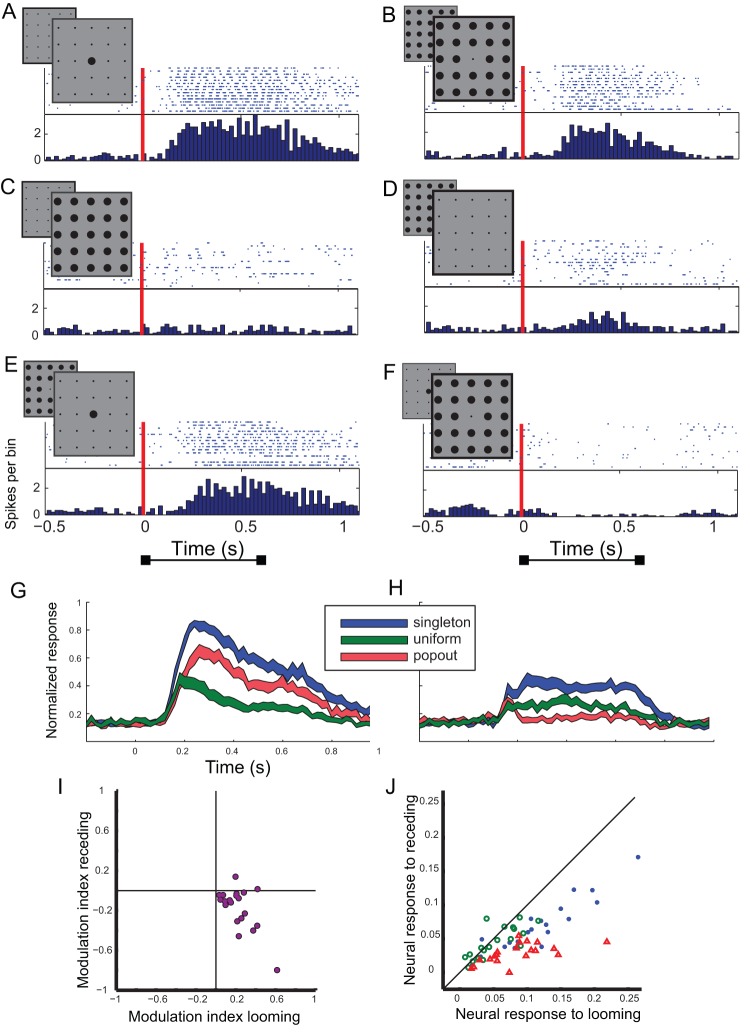
Summary of results from the looming test. A–F) The responses of a single site to the looming/receding test. A) The raster plot shows responses to 15 repetitions of a looming dot inside the RF while the background dots are static (singleton condition). The inset demonstrates the visual stimulus. The hind frame illustrates the initial picture, at the onset of stimulation, and the front frame illustrates the final picture. The corresponding PSTH is shown below the raster plot. The red vertical line designates the onset of stimulation, i.e., the initiation of movement. B) Responses to 15 repetitions of a receding dot in the RF while the background dots are static (singleton condition). Format as in A. C) Responses to 15 repetitions of all dots looming together (uniform condition). Format as in A. D) Responses to 15 repetitions of all dots receding together (uniform condition). Format as in A. E) Responses to 15 repetitions of the center dot looming together with the background dots receding (contrasting condition). Format as in A. F) Responses to 15 repetitions of the center dot receding together with the background dots looming (contrasting condition). Format as in A. G) The PSTH curve of the population response to the singleton looming (blue curve) is compared with the PSTH curve of the population response to the uniform looming (green curve) and with the PSTH curve of the population response to the contrasting looming (red curve). The width of the curves designate the SEM. H) The PSTH curve of the population response to the singleton receding (blue curve) is compared with the PSTH curve of the population response to the uniform receding (green curve) and with the PSTH curve of the population response to the contrasting receding (red curve). The width of the curves designate the SEM. I) The scatterplot shows the modulation indices for the receding center stimulus versus the looming center stimulus. Each dot represents results from a single recording site. J) The neural responses to the receding stimuli are shown versus the responses to the looming stimuli. Each dot represents results from a single site. Blue symbols show results from the singleton conditions, red from the contrasting conditions and green symbols results from the uniform conditions. The diagonal line displays the locus of equal responses.

The looming paradigm was tested in 20 recording sites. One typical example is shown in figure 5A-F. This site responded rigorously to both looming and receding stimuli in the singleton conditions (Fig 5A and B, respectively). However, the response to the looming dot was significantly larger than the response to the receding dot (*t*-test, p<0.05). In the uniform condition the response to the looming dot was completely suppressed compared to the singleton condition (Fig 5C). The response to the uniform receding dot was also highly suppressed (t-test <0.05, Fig 5D) but to a less extent. In contrast, the response to the looming stimulus in the contrasting condition was only slightly suppressed compared to the singleton context (t-test <0.05, Fig 5E) resulting with a much stronger response to the contrasting stimulus compared to the uniform. However, this was not the case when the target was receding. The response to the receding contrasting stimulus was completely suppressed (Fig 5F) even more than the uniform receding stimulus (Fig 5D). Thus, a looming background induced much stronger inhibition in the RF compared to a receding background resulting with positive contrast sensitivity to the looming stimulus inside the RF and negative contrast sensitivity to the receding stimulus inside the RF. This behavior was apparent at the population responses as well. Figure 5G shows the population PSTHs to the looming stimulus inside the receptive field. The looming stimulus in the contrasting condition yielded an average response (red curve) larger than the uniform condition (green curve). This trend was reversed when the receding stimulus was inside the RF (Fig 5H). In this case the uniform condition (green curve) yielded larger average response compared to the contrasting condition (red curve).

Figure 5I shows a scatter plot of the modulation indices. Most dots were in the lower right quadrant (18 out of 20, Chi-square <0.01), indicating contrast enhancement when the stimulus inside the RF was looming but contrast suppression when the stimulus was receding. Figure 5J shows a scatterplot of the responses to the receding stimulus versus the responses to the looming stimulus, in the singleton (blue dots), uniform (green dots) and the contrasting (red dots) conditions. Most recording sites responded stronger to the singleton looming stimulus compared to the singleton receding (sign test, p<0.05). On the other hand in the uniform condition the dots were scattered evenly across the equality-line (sign test, p>0.05). In the contrasting conditions, like in the singleton condition, most of the sites responded stronger to the looming stimulus (sign test, p<0.05).

## Discussion

### Saliency and competition in the OT

The results of this study show that in the space map of the OT, background stimuli outside of the receptive field can strongly suppress or eliminate responses to stimuli inside the receptive field. The responses to the singleton conditions were stronger from the uniform and contrasting conditions in all test paradigms (orientation, motion and looming). This robust inhibition from the surround is consistent with previous studies in the OT of birds which report global inhibitory interactions across a large region of the space map [10], [23], [24]. This global inhibition enables the more powerful stimulus to suppress responses to the less powerful stimulus and by thus give rise to competitive interactions [11]. Feedback circuitry involving the cholinergic and GABAergic connections from the nearby nucleus isthmi has been suggested to perform this competitive interaction [12], [24]–[26].

In the OT or its mammalian homologue SC most neurons are selective to the location and intensity of the stimulus but to a much lesser extent to other sensory features such as orientation, motion direction, frequency and the modality [19], [27]–[29]. Although some selectivity to motion direction [30] and orientation [31] exists, there is no evidence for functional organization of these sensory features. Moreover, tectal neurons habituate strongly to repeated stimuli [17], [32], [33]. This, together with the global inhibition mentioned above, suggest that the OT represents the location of salient stimuli based on novelty and intensity. However, the perceived saliency of a stimulus is strongly affected by how different it is from its surround [1], [34]. It is an open question to what extent contrasting stimuli are represented in the OT.

### Orientation paradigm

In the orientation paradigm both uniform and contrasting conditions resulted with a similar strong suppression of the responses to the bar inside the receptive field. This finding, is in contrast to the results of the behavioral experiments in barn owls which showed that orientation difference between a target and its background is sufficient to enhance the perceived saliency of the target [5]. One possibility to explain the discrepancy is that the stimulus used in this study is not adequate. Here we flashed the orientation display on the screen for a relatively short period whereas in free viewing barn owls the stimulus is continuously displayed and the barn owls actively explore it. Another possibility is that that the computation and representations of the orientation contrast is happening somewhere else in the brain. The brain circuitry that computes orientation contrast is likely to include at some point orientation sensitive neurons [35]. The visual Wulst in barn owls is a forebrain area that, unlike the OT, contains orientation sensitive neurons [36]–[38] and is considered equivalent to V1 [39]. Selectivity to bars oriented differently from the background has been shown to exist in some V1 neurons [40]. It is therefore worth testing if neurons in the visual Wulst are selective to orientation contrasting stimuli and consequently may underlie the exclusive behavioral responses to differently oriented targets.

### Looming/receding paradigm

The results of the looming paradigm were markedly different from the orientation paradigm. The responses to the looming stimulus inside the RF was substantially suppressed when the background elements were also looming but when they were receding the suppression was reduced, giving rise to apparent contrast sensitivity. However, this was not a reflection of general sensitivity to contrast because no enhanced responses were observed when the center was a receding stimulus in a looming background (Fig 5H). This result can be explained by the notion that the responses are determined by the relative strengths of the stimuli inside and outside of the RF. The looming stimulus seems to be an inherently stronger stimulus compared to the receding stimulus (Fig 5J). This inherent perceptual difference, which probably reflects the biological importance of an abrupt detection of a looming stimulus, is common in different species [41]–[44]. Thus, if the stimulus inside the RF is receding and the stimuli outside are looming the strength of the outside stimuli overtakes, giving rise to strong suppression even though the stimulus is a contrasting stimulus.

### Motion paradigm

In the motion paradigm the uniform conditions gave rise to clear suppressions of the responses, as in the orientation and looming paradigms. However, the contrasting conditions gave rise to a different result. Here the responses to both directions of motion were stronger than in the uniform condition. Such a response cannot be explained by the notion mentioned above that the responses of tectal neurons reflect the relative strength of the competing stimuli. One motion direction is not a stronger stimulus than another motion direction. Therefore, there is no reason to expect that the responses in the contrasting conditions, to both directions, will be less suppressed compared to the uniform conditions.

Importantly, motion direction contrast sensitivity has been reported in tectal neurons of pigeons [23], [45] and monkeys [46]. The paradigm used by these authors was a moving target on top of a moving textured pattern. Maximal response suppression was achieved when the difference between the direction of movement inside the receptive field and the movement of the background was small. As in our study (Fig 4), the sensitivity to relative motion occurred even for large separation range between the target and backgrounds [30], [46]. Thus, together with our findings from the barn owl, it seems that this property is not a specialization of a certain species and reflects a general tendency of tectal neurons to report the movement inside their RF relative to global motion outside of the receptive field. One noteworthy difference between our results and previous results in pigeons is that in pigeons when the background moved in the opposite direction to the center the neural responses were mostly facilitated compared to the center motion alone [23], [45], suggesting double-opponency RFs [30]. In our experiments the responses to the center stimulus in the singleton condition were always larger compared to the contrasting condition. In this aspect our results resemble more the results from the monkey SC were no facilitation but rather less inhibition was reported [46]. This is an important difference because it means that the neurons do not code the velocity contrast between the background elements and the object but rather respond to localize motion.

The property to respond stronger to object motion that is in the opposite direction of the background may be used for the rapid detection of localized movement, a property that is consistent with the proposed role of the OT in the selection of the most salient stimulus. However, for the same reason one would expect that tectal neurons will respond specifically to local orientation as well, whereas, the results of this study suggest that they don't. Why it is that motion is uniquely processed in the OT? One possibility is that the sensitivity to motion contrasts is important to avoid self-induced motion responses [47]. Moreover, motion is a strong cue for figure-ground segregation playing an important role in camouflage breaking [48]. The importance of motion cues in tectal responses of pigeons has been demonstrated by using kinematograms as stimuli [49]. Neurons responded to objects emerging from the coherent motion of sets of dots when the “objects” moved opposite from the background. When the emerging objects moved with the background (the so called “hole” configuration) tectal responses were inhibited even though the dots moved in the opposite direction. This demonstrates that it is the motion of the emerging figure that determines the neural responses and not any other feature.

### Relation of results to pop-out

The result that tectal neurons are selective to the motion contrasting stimulus relative to the uniform motion stimulus is consistent with the idea that motion pop-out is expressed in tectal neurons. However, such a result does not necessarily imply genuine pop-out sensitivity. Not all cases in which disparities exist between the center and the surround are perceived as pop-outs. For pop-out to take place the background needs to be uniform in at least one feature that is different from the center [34], [50]. To test for genuine pop-out sensitivity, conjunction stimuli in-which the center is different from the surround by a unique conjunction of features, are useful [51], [52]. In conjunction stimuli the target is different from its surround but it does not pop-out. Therefore, neurons that are genuinely selective for pop-out stimuli should be more sensitive to contrasting stimuli compared to conjunction as well as uniform stimuli [52]. Since in this study, like in previous studies in the avian OT [23], [30], [45], only uniform conditions were compared it is still an open question if tectal neurons respond to motion pop-outs per-se. One experimental difficulty to perform the above mentioned control is that for conjunction stimuli, at least two visual features are needed (e.g. motion direction and bar orientation), however, in the optic tectum contrast sensitivity of visual features, apart for motion, were either not studied or explicitly shown not to bear specific effects (Fig. 2).

### Mechanisms for the preferred responses to the motion contrasting stimuli

Neurons that are specifically sensitive to local motion within their RF have been found in the retina of rabbit and salamander [53]. It has been suggested that such neurons segregate object motion from optic flow and participate in motion pop-out effects [53]. Hence, the basics for the computation of motion contrast selectivity in the OT may reside in the retina. However, the extent of the lateral inhibition region in the OT seems greater than what has been reported in the retina. Responses to object motion were suppressed by same motion elements 20 degrees away from the center (Fig 4). Other studies reported lateral inhibition from same stimulus elements presented 100 degrees from the RF [10], [30], whereas in retinal ganglion cells the extent of the surround suppression was about 3 times the radius of the classical RF (Fig. 3A in [53]). Moreover, the retinal cells seemed specialized to cope with the special optic flow induced by the “random-walk” of fixational eye movements [53] and not with synchronized center and background motion. Therefore it is likely that additional mechanisms outside of the retina take place. The long range effects observed here and in other studies may imply that the global inhibition network of the tecto-isthmi loop [10], [24] is involved in generating the motion contrast effects. This requires the global inhibition to be specific to the direction of motion. However, the global inhibitory network from the Imc is activated by precise topographic connections from the OT and the Ipc [13], [54]. This anatomical organization is suited to process spatial information and not feature-specific information [25].

An alternative explanation is that top-down connections from the forebrain play a role in mediating the sensitivity to local motion. In this respect, the arcopallium gaze fields (AGF), a forebrain area which is considered equivalent to the frontal eye fields in primates [55], modulates tectal responses in a space-specific manner. Microstimulations in the AGF enhanced visual and auditory responses to stimuli within the RF of the microstimulation site and suppressed responses to stimuli outside the RF [56]. It is possible that the location of local motion is first computed in forebrain circuitry giving rise to top-down signals that specifically increase the gain to stimuli in the location of the target. Supporting this hypothesis is the study by Davidson et al. [57] which showed that inactivation of cortical inputs to the SC reduced the sensitivity to relative motion. Interestingly, the AGF sends axons both to the OT and the Ipc [58]. Therefore it is possible that the tecto-isthmi circuitry is controlled by the forebrain to mediate tectal responses to motion contrasting stimuli.
